# Management by the intensivist of gastrointestinal bleeding in adults and children

**DOI:** 10.1186/2110-5820-2-46

**Published:** 2012-11-09

**Authors:** David Osman, Michel Djibré, Daniel Da Silva, Cyril Goulenok

**Affiliations:** 1 AP-HP, Hôpitaux universitaires Paris-Sud, Hôpital de Bicêtre, Service de réanimation médicale, Le Kremlin-Bicêtre, F-94270, France; 2 AP-HP, Hôpitaux universitaires Est Parisien, Hôpital Tenon, Service de pneumologie et réanimation, Paris, F-75020, France; 3 Hôpital Delafontaine, Service de réanimation polyvalente, Saint-Denis, F-93200, France; 4 Hôpital Privé Jacques Cartier, Service de réanimation polyvalente, Massy, F-91300, France

**Keywords:** Gastrointestinal bleeding, Intensive care, Ulcer, Gastric/esophageal varices, Recommendations

## Abstract

Intensivists are regularly confronted with the question of gastrointestinal bleeding. To date, the latest international recommendations regarding prevention and treatment for gastrointestinal bleeding lack a specific approach to the critically ill patients. We present recommendations for management by the intensivist of gastrointestinal bleeding in adults and children, developed with the GRADE system by an experts group of the French-Language Society of Intensive Care (Société de Réanimation de Langue Française (SRLF), with the participation of the French Language Group of Paediatric Intensive Care and Emergencies (GFRUP), the French Society of Emergency Medicine (SFMU), the French Society of Gastroenterology (SNFGE), and the French Society of Digestive Endoscopy (SFED). The recommendations cover five fields of application: management of gastrointestinal bleeding before endoscopic diagnosis, treatment of upper gastrointestinal bleeding unrelated to portal hypertension, treatment of upper gastrointestinal bleeding related to portal hypertension, management of presumed lower gastrointestinal bleeding, and prevention of upper gastrointestinal bleeding in intensive care.

## Review

### Introduction

Intensivists are regularly confronted with the question of gastrointestinal (GI) bleeding. They notably need to manage acute GI bleeding, a frequent and severe condition [1], the mortality of which has probably not changed for 20 years [1] but could be reduced through recent diagnostic and therapeutic advances [2]. They also should define, for each patient admitted to intensive care, how to prevent upper GI bleeding related to “stress ulceration”. a now uncommon complication [3], but for which drug prophylaxis is widely used [4] and often involves proton pump inhibitors (PPIs) [4]. Because the latest international recommendations for the management of acute nonvariceal upper GI bleeding [2] or for GI bleeding related to portal hypertension [5] lack a specific approach to severe forms, and because the French consensus conference on prevention of “stress-related” upper GI bleeding in intensive care was nearly 25 years ago [6], we decided to draw up these recommendations (Table 1).

**Table 1:** Recommendations for management by the intensivist of gastrointestinal bleeding

					
**Area 1**: Management of GI bleeding before endoscopic diagnosis		11	In children, in massive hematochezia and/or with hemodynamic consequences, and when GI endoscopic findings are normal, an emergency scintigraphy to search for a Meckel’s diverticulum should be used and/or a surgical exploration (McBurney’s incision or coelioscopy) (*strong agreement*)	5	In the presence of stigmata associated with a high risk of rebleeding (Forrest type Ia, Ib, IIa, IIb), PPI treatment should be continued at “high” doses for 72 h *(strong agreement)*
1	Nasogastric intubation may help confirm, but cannot discount, suspected upper GI bleeding (*strong agreement*)	12	Vasoactive treatment (terlipressin or somatostatin derivative) should be administered as soon as possible when portal hypertension is the suspected cause if GI bleeding (*strong agreement*)	6	Second-look EGD should not be done routinely *(strong agreement)*
2	Suspected rupture of esophageal/gastric varices probably does not contraindicate nasogastric intubation (*strong agreement*)	13	In a patient already treated with noradrenaline, specific vasoactive treatment of the splanchnic area (terlipressin, somatostatin, somatostatin derivative) should probably be administered when portal hypertension is the suspected cause of GI bleeding *(expert opinion, weak agreement).*	7	Second-look EGD should probably be done when a high-risk stigmata has been observed*(weak agreement)*
3	To ensure emptying of the stomach content before EGD, intravenous erythromycin should be administered at a dose of 250 mg (5 mg/kg in children), in the absence of contraindications (*strong agreement*)	14	Specific vasoactive treatment of the splanchnic area (terlipressin, somatostatin, somatostatin derivative) should probably not be administered when portal hypertension is not the suspected cause of GI bleeding *(weak agreement)*	8	Patients with ulcer bleeding should not be treated with H_2_ receptor antagonists *(strong agreement)*
4	If a nasogastric tube has been inserted, gastric lavage to empty the stomach is an alternative to administration of erythromycin (*weak agreement*)	15	In GI bleeding potentially caused by ulcers, PPI treatment should be started without waiting for endoscopic diagnosis *(weak agreement)*	9	In adults, in case of Forrest type Ia and Ib, first- intention selective arterial embolization by interventional radiology should probably be used following failure of endoscopic therapy *(weak agreement)*
5	In adults, the Rockall score and the Glasgow-Blatchford bleeding score can probably help to identify patients at high risk of morbidity and mortality and to refer them to an intensive care unit (*strong agreement*)	16	In GI bleeding potentially caused by ulcers, high-dose PPI treatment should probably be administered *(weak agreement)*	10	In adults, in case of Forrest type Ia and Ib and catastrophic bleeding, first-intention surgical hemostasis should probably be used following failure of endoscopic therapy if local conditions do not allow arterial embolization*(strong agreement)*
6	EGD should be done in the 24 h following the admission of the patient with suspected upper GI bleeding (*strong agreement*)	**Area 2**: Treatment of upper GI bleeding unrelated to portal hypertension		11	Biopsy screening for *Helicobacter pylori* infection can be performed during the first EGD for GI bleeding without worsening the bleeding *(strong agreement)*
7	EGD should be probably done in the b12 h following the admission of the patient with suspected esophageal/gastric variceal bleeding (*strong agreement*)	1	In the presence of stigmata associated with a low risk of rebleeding (Forrest type IIc and III), endoscopic hemostasis should not be used *(strong agreement)*	12	There is probably no advantage to emergency treatment of *Helicobacter pylori* infection in the case of GI ulcer bleeding *(strong agreement)*
8	EGD should be probably done as soon as possible, and once the patient is resuscitated, when active upper GI bleeding is suspected (*strong agreement*)	2	In the presence of stigmata associated with a low risk of rebleeding (Forrest type IIc and III), PPI treatment at “standard” doses should be continued *(strong agreement)*	13	Aspirin antiplatelet therapy should probably be maintained in the case of GI ulcer bleeding until consultation with specialists *(weak agreement)*
9	In massive hematochezia and/or hemodynamic consequences, an EGD should be performed as soon as possible (*strong agreement*)	3	In the presence of stigmata associated with a high risk of rebleeding (Forrest type Ia, Ib, IIa), endoscopic hemostasis should be performed *(strong agreement)*	14	In dual antiplatelet therapy, clopidogrel should probably be stopped in the case of ulcer bleeding until consultation with specialists *(strong agreement)*
10	In adults, in massive hematochezia and/or with hemodynamic consequences, a CT angiography should be performed in emergency, if EGD is not rapidly available and/or if an aortoenteric fistula is suspected (*strong agreement*)	4	In the presence of an adherent clot (Forrest type IIb), endoscopic hemostasis is possible when the clot is small *(strong agreement)*	**Area 3**: Treatment of upper GI bleeding related to portal hypertension	
1	Endoscopic therapy of bleeding esophageal/gastric varices should be done during initial EGD *(strong agreement)*	13	Blood transfusion in most patients should probably target a hemoglobin concentration of 7 to 8 g/dL *(strong agreement)*	5	When lower GI bleeding is massive, surgical hemostasis should be proposed in case of arterial embolization or colonoscopy failure or rebleeding *(strong agreement)*
2	Endoscopic therapy of bleeding esophageal varices is based on band ligation. Sclerotherapy is an alternative in the very young child *(strong agreement)*	14	In GI bleeding in patients with cirrhosis, there is probably no indication for administration of fresh-frozen plasma with the objective to correct a coagulopathy *(strong agreement)*	6	When lower GI bleeding is catastrophic, surgical hemostasis should be performed if arterial embolization is not possible under local conditions *(strong agreement)*
3	Endoscopic therapy of bleeding gastric varices is based on obturation with tissue adhesives *(strong agreement)*	15	In GI bleeding in patients with cirrhosis, there is no indication for administration of fresh-frozen plasma before EGD *(strong agreement)*	7	In massive or persistent lower GI bleeding, the small intestine should be examined as soon as possible when CT angiography and colonoscopy fail to locate the source of bleeding *(strong agreement)*
4	Vasoactive treatment should be continued for 3 to 5 days after endoscopic therapy of esophageal/gastric varices rupture *(strong agreement)*	16	In GI bleeding in patients with cirrhosis, there is no indication for the administration of factor VIIa *(strong agreement)*	**Area 5**: Prevention of upper GI bleeding in intensive care	
5	In adults, after endoscopic hemostasis of bleeding related to portal hypertension, the placement of a TIPS within 72 h should be considered in high-risk patients *(strong agreement)*	17	In GI bleeding in patients with cirrhosis, platelet transfusion should probably be considered when bleeding is uncontrolled and platelet count is <30,000/mm^3^*(strong agreement)*	1	Patients with a history of peptic ulcer admitted to intensive care should probably be considered at risk of GI bleeding *(strong agreement)*
6	Balloon tamponade should be considered after endoscopy failure pending radical treatment of portal hypertension. In child, its use should probably be envisaged if emergency EGD is not possible *(strong agreement)*	18	In adults, in esophageal/gastric bleeding, beta-blocker treatment should be started when vasoactive treatment is discontinued *(strong agreement)*	2	Early enteral feeding is effective in preventing “stress ulcer” bleeding *(strong agreement)*
7	Antibiotic prophylaxis with third-generation cephalosporin or with fluoroquinolone for 5 to 7 days should be given to any cirrhotic patient with GI bleeding *(strong agreement)*	19	After ligation of esophageal varices, nasogastric intubation should probably be avoided *(expert opinion, strong agreement)*	3	Patients requiring mechanical ventilation for more than 48 h and for whom enteral feeding is not possible should be considered to be at risk of “stress ulcer” bleeding *(strong agreement)*
8	Lactulose treatment to prevent hepatic encephalopathy should probably not be initiated during GI bleeding in a cirrhotic patient (*expert opinion, strong agreement*)	**Area 4**: Management of presumed lower GI bleeding		4	Patients admitted to intensive care with kidney failure and/or coagulopathy and/or receiving antiplatelet therapy should be considered to be at risk of “stress ulcer” bleeding *(strong agreement)*
9	In adults, PPI therapy should not be initiated or continued when EGD has confirmed a diagnosis of ruptured esophageal/gastric varices (*strong agreement*)	1	In adults with massive hematochezia, demonstration of active bleeding by abdominal CT angiography or arteriography justifies embolization as first-line therapy *(strong agreement)*	5	Routine drug prophylaxis of “stress ulcer” should not be used in intensive care patients with enteral feeding *(strong agreement)*
10	In children, PPI therapy should probably be initiated or continued in case of esophageal/gastric varices rupture *(weak agreement)*	2	In massive hematochezia, and in the absence of detectable bleeding on CT angiography or arteriography a prepared colonoscopy should be performed within 24 h *(strong agreement)*	6	Ulcer prophylaxis medication should probably be given routinely in intensive care patients with a history of peptic ulcer (even if enterally fed) *(weak agreement)*
11	One objective of hemodynamic treatment during esophageal/gastric varices rupture should be to restore a satisfactory mean blood pressure to preserve tissue perfusion *(strong agreement)*	3	In massive and persistent hematochezia, and in the absence of detectable bleeding on abdominal CT angiography or arteriography, a prepared colonoscopy should probably be done within 12 h with the objective of performing endoscopic hemostasis *(strong agreement)*	7	Ulcer prophylaxis medication should probably be given routinely to intensive care patients receiving antiplatelet therapy (even if enterally fed) *(weak agreement)*
12	In adults, during esophageal/gastric varices rupture, early hemodynamic treatment should probably maintain mean blood pressure at approximately 65 mmHg in most patients *(strong agreement)*	4	In adults with massive hematochezia, rectosigmoidoscopy should probably be done if full colonoscopy cannot be performed within 24 h *(strong agreement)*	8	In the absence of enteral feeding, ulcer prophylaxis medication should probably be given to ventilated patients *(weak agreement)*
9	In the absence of enteral feeding, ulcer prophylaxis medication should probably be given to patients with coagulopathy *(weak agreement)*	13	A large bore nasogastric tube for aspiration should probably be replaced by a small-calibre enteral tube as soon as possible *(expert opinion, strong agreement)*		
10	In children, a pediatric risk of mortality score (PRISM) > 10 associated with respiratory failure or coagulopathy or both probably calls for ulcer prophylaxis *(strong agreement)*	14	Antacids should not be used to prevent “stress ulcer” bleeding *(strong agreement)*		
11	Screening for *Helicobacter pylori* should not be routine in intensive care patients *(strong agreement)*	15	H_2_ receptor antagonists and PPIs are probably comparable but of low efficacy in preventing “stress ulcer” bleeding *(weak agreement)*		
12	A nasogastric tube should probably be removed once it is no longer used *(expert opinion, strong agreement)*	16	H_2_ receptor antagonists and PPIs are probably comparable regarding the risk ventilator associated pneumonia during mechanical ventilation *(strong agreement)*		

### Methodology

The SRLF brought together a group of experts representing the various disciplines involved in the management of severe GI bleeding (critical care, hepatogastroenterology, emergency care, radiology, surgery, pediatrics) to draw up these recommendations. The organizing committee and the coordinator first defined the questions to be considered and designated the experts responsible for each question. Using the GRADE system (Grading of Recommendations, Assessment, Development and Evaluation) [7,8], the literature was analyzed and recommendations were formulated. A level of proof was defined for each literature reference according to the type of study, and could be reevaluated taking into account the methodological quality of the study. The literature references common to each assessment criterion were then grouped. An overall level of proof was determined for each assessment criterion taking into account the levels of proof of each literature reference, the consistency of the results between the different studies, the cost-effectiveness analysis, the directness of the evidence, etc. A “strong” level of proof enabled a “strong” recommendation (“must be done,” “must not be done…”). A “moderate,” “weak,” or “very weak” level of proof resulted in a “conditional” recommendation (“should probably be done”, “should probably not be done…”). The proposed recommendations were presented and discussed one by one. The aim was not necessarily to reach a single and convergent opinion for all proposals, but rather to highlight points of agreement, and points of disagreement or indecision. Each recommendation was then scored by each of the experts on a scale of 1 (complete disagreement) to 9 (complete agreement). The collective scoring was established using the RAND/UCLA appropriateness method [9]: after elimination of the extreme values (outliers), the median and confidence interval of the individual scores were calculated. The median defined agreement between the experts when it was between 1 and 3, disagreement between 7 and 9 and indecision between 4 and 6. The agreement, disagreement, or indecision was “strong” if the confidence interval was within one of three ranges: (1–3), (4–6), or (7–9) and “weak” if the confidence interval straddled two ranges. In the absence of strong agreement, the recommendations were reformulated and again scored with a view to achieving a better consensus. Three rounds of scoring were therefore performed.

### Area 1: Management of GI bleeding before endoscopic diagnosis

1.Nasogastric intubation may help confirm, but cannot discount, suspected upper GI bleeding *(strong agreement).*

Upper GI bleeding should always be diagnosed by esophagogastroduodenoscopy (EGD) [2,10]. Pending endoscopy, insertion of a nasogastric tube can guide the diagnostic approach, notably in a setting of acute anaemia or hemorrhagic shock. Three recently compiled retrospective analyses assessed the capacity of nasogastric aspiration to differentiate an upper from a lower source of GI bleeding [11]. Apart from one study, the design of which probably generated a high proportion of false positives, insufficient negative predictive values were reported (61%; 95% CI 53–68 and 64%; 95% CI 43–80), albeit associated with very satisfactory positive predictive values (81%; 95% CI 69–90 and 93%; 95% CI 81–98) [11].

2.Suspected rupture of esophageal/gastric varices probably does not contraindicate nasogastric intubation *(strong agreement).*

The hemorrhagic risk associated with nasogastric tube insertion when rupture of esophageal varices is suspected is unknown. In a population admitted to hospital because of rupture of esophageal varices in approximately 35% of cases, gastric lavage after nasogastric tube placement was compared with intravenous erythromycin for GI endoscopy preparation [12]. There was no between-group difference in rebleeding frequency or in the number of transfused blood units [12].

3.To ensure emptying of the stomach content before EGD, intravenous erythromycin should be administered at a dose of 250 mg (5 mg/kg in children), in the absence of contraindications *(strong agreement).*

4.If a nasogastric tube has been inserted, gastric lavage to empty the stomach is an alternative to administration of erythromycin *(weak agreement).*

In a recent meta-analysis, the use of a prokinetic agent (erythromycin or metoclopramide) was associated with reduced need for a repeat EGD (odds ratio [OR] 0.55; 95% CI 0.32-0.94) [13].In contrast, there was no improvement in the length of hospital stay, the need for surgery, or the number of transfused blood units [13]. Erythromycin is useful in bleeding ulcer and in bleeding associated with portal hypertension [14,15]. Last, a recent study found no difference between erythromycin, gastric lavage, and the two combined in term of conditions for endoscopy [12]. Nasogastric intubation was, however, often poorly tolerated and caused pain [12].

5.In adults, the Rockall score and the Glasgow-Blatchford bleeding score can probably help to identify patients at high risk of morbidity and mortality and to refer them to an intensive care unit *(strong agreement*).

The Rockall score and the Glasgow-Blatchford bleeding score, which have been validated in many studies [16,17], were developed for early identification of high-risk patients needing intensive care and of low-risk patients who can be discharged early. The Rockall score (Table 2) comprises clinical, biochemical, and endoscopic data and is correlated with the risk of rebleeding [18]. The Glasgow-Blatchford bleeding score (Table 3) includes clinical and biochemical data, but not EGD, and is useful to predict the need for intervention (hospitalization, transfusion, surgery) or death [19].

6.EGD should be done in the 24 h following the admission of a patient with suspected upper GI bleeding *(strong agreement).*

7.EGD probably should be done in the 12 h following the admission of the patient with suspected esophageal/gastric variceal bleeding *(strong agreement).*

8.EGD probably should be done as soon as possible, and once the patient is resuscitated, when active upper GI bleeding is suspected *(strong agreement).*

**Table 2:** Rockall score

**Score**	**0**	**1**	**2**	**3**
Age (yr)	<60	60-70	>80	
Shock	No shock	Pulse >100		
		SBP >100 mmHg		
Comorbidity	No	No	Ischemic heart disease	Renal failure
			Cardiac failure	Liver failure
			Major comorbidity	Disseminated malignancy
Diagnosis	Mallory Weiss tear or	All other diagnoses	Gastrointestinal malignancy	
	No lesion			
Evidence of bleeding	None or		Blood	
	Dark spot		Visible or spurting vessel	
			Adherent clot	

A total score less than 3 carries good prognosis and total score more than 8 carries high risk of mortality.

SBP, systolic blood vessel.

From Rockall TA, Gut 1996.

**Table 3:** Glasgow-Blatchford score

**Admission risk marker**		**Score**
Blood urea (mmol/l)	≥6.5 et < 8	2
	≥8 et < 10	3
	≥10 et < 25	4
	≥25	6
Hemoglobin (g/L) for men	≥12 et < 13	1
	≥10 et < 12	3
	<10	6
Hemoglobin (g/L) for women	≥10 et < 12	1
	<10	6
Systolic blood pressure (mmHg)	≥100 et < 109	1
	≥90 et < 100	2
	<90	3
Other markers	pulse ≥100	1
	Melena	1
	Syncope	2
	Hepatic disease	2
	Cardiac failure	2

A total score more than 8 carries high risk justifying ICU admission.

From Blatchford O, Lancet 2000.

EGD within 24 h after admission of a patient with suspected upper GI bleeding reduces transfusion requirement, second-look endoscopy, and emergency surgery [2,10,20]. However, the influence of this 24-h interval on mortality remains controversial [2,10,20]. Earlier EGD (within 6 to 12 h) is subject to debate. A meta-analysis of three randomized trials [2] showed no benefit of early endoscopy in terms of mortality, rebleeding, or need for surgery. In high-risk patients, however, early endoscopy may prove valuable. One study showed that early endoscopy in patients with blood in gastric aspirates was associated with reduced transfusion requirement and reduced hospital stay [21]. Another study identified independent predictors of benefits of early EGD that included fresh blood in gastric aspirates, hemodynamic instability, and hemoglobin <8 g/dL [22]. A recent study showed that when the Glasgow-Blatchford score was ≥12, the mortality of patients in whom endoscopy was performed within the first 13 h was lower than in those who underwent endoscopy after 13 h [23]. Lastly, when esophageal/gastric variceal bleeding is suspected, EGD in the first 12 h is usually recommended [5,24].

9.In massive hematochezia and/or with hemodynamic consequences, an EGD should be performed as soon as possible *(strong agreement).*

Approximately 10% of cases of massive hematochezia are related to upper GI bleeding, thus justifying EGD before colonoscopy [25]. When the upper GI endoscopic findings are normal, the question sometimes arises whether to repeat EGD before colonoscopy. EGD need not be repeated if no blood is found in the upper GI tract on a first examination performed in good conditions [26].

10.In adults, in massive hematochezia and/or with hemodynamic consequences, an abdominal CT angiography should be performed in emergency, if EGD is not rapidly available and/or if an aortoenteric fistula is suspected *(strong agreement)*.

Several studies have shown the great value of abdominal CT angiography in topographic diagnosis of upper or lower GI bleeding [27-29]. Improved CT angiography techniques also can be used for etiological diagnosis and to guide treatment [29,30]. CT angiography enables timely recognition of the rare aortoenteric fistulas [31].

11.In children, in massive hematochezia and/or with hemodynamic consequences, and when GI endoscopic findings are normal, an emergency scintigraphy to search for a Meckel’s diverticulum should be used and/or a surgical exploration (McBurney’s incision or coelioscopy) (*strong agreement*).

In children, massive hematochezia is infrequent and mostly caused by variceal rupture in a setting of portal hypertension or Meckel’s diverticulum. More rarely, vascular malformations of the colon or small intestine are observed [32]. If the GI endoscopic findings are normal, thus discounting rupture of varices, scintigraphy or even surgical exploration should be performed to search for a Meckel’s diverticulum [33].

12.Vasoactive treatment (terlipressin or somatostatin or a somatostatin derivative) should be administered as soon as possible when portal hypertension is the suspected cause of GI bleeding *(strong agreement).*

Vasoactive medication will stop bleeding in 75% to 80% of cases [24]. A literature analysis suggests that once portal hypertension is clinically suspected, including at the prehospital management phase, vasoactive medication improves the quality of patient transport and then of the EGD [5,34]. Two drug classes are available: vasopressin derivatives (in particular terlipressin) and synthetic derivatives of somatostatin (octreotide, vapreotide). Comparison of somatostatin with terlipressin showed similar first bleeding, rebleeding, and mortality rates [35]. Combined with endoscopic therapy, octreotide significantly reduces rebleeding in comparison with placebo [36-38]. Lastly, octreotide was better than vasopressin [39] and equivalent to terlipressin [40].

13.In a patient already treated with noradrenaline, specific vasoactive treatment of the splanchnic area (terlipressin, somatostatin, somatostatin derivative) should probably be administered when portal hypertension is the suspected cause of GI bleeding *(expert opinion, weak agreement).*

14.Specific vasoactive treatment of the splanchnic area (terlipressin, somatostatin, somatostatin derivative) should probably not be administered when portal hypertension is not the suspected cause of GI bleeding *(weak agreement).*

A 1997 meta-analysis showed that somatostatin treatment had benefits in a setting of upper GI bleeding unrelated to portal hypertension [41]. The comparison was, however, made with an H_2_ receptor antagonist and a placebo [41] and no study has examined this question since the advent of PPIs.

15.In GI bleeding potentially caused by ulcers, PPI treatment should be started without waiting for endoscopic diagnosis *(weak agreement).*

16.In GI bleeding potentially caused by ulcers, high-dose PPI treatment should probably be administered *(weak agreement).*

A recent meta-analysis showed that PPIs at “standard” doses (compared with no treatment, placebo, or an H_2_ receptor antagonist) administered beforehand facilitated EGD by reducing the proportion of patients with stigmata of recent hemorrhage or active bleeding and by reducing requirement for endoscopic therapy [42]. One study showed that before endoscopic therapy, the administration “high” PPI doses, compared with placebo, reduced transfusion requirement, rebleeding, and hospital stay [43]. This question has not been examined by comparing “high” and “standard” doses.

### Area 2: Treatment of upper GI bleeding unrelated to portal hypertension

1.In the presence of stigmata associated with a low risk of rebleeding (Forrest type IIc and III), endoscopic hemostasis should not be used *(strong agreement).*

The natural history of ulcer disease shows a rebleeding rate of approximately 5% only in the case of Forrest type IIc or III (Table 4) [44].

2.In the presence of stigmata associated with a low risk of rebleeding (Forrest type IIc and III), PPI treatment at “standard” doses should be continued *(strong agreement).*

**Table 4:** Forrest classification

	**Class**	**Risk of rebleeding**
Clean base	III	low
Hematin covered flat spot	IIc	low
Adherent clot	IIb	high
Visible vessel	IIa	high
Oozing hemorrhage	Ib	high
Spurting hemorrhage	Ia	high

From Laine L, New Engl J Med 1994.

PPIs at “standard” doses have proven effective in peptic ulcer and recent French recommendations reiterate that there is no difference between the five available PPIs (esomeprazole, lansoprazole, omeprazole, pantoprazole, rabeprazole) [45].

3.In the presence of stigmata associated with a high risk of rebleeding (Forrest type Ia, Ib, IIa), endoscopic hemostasis should be performed *(strong agreement).*

A meta-analysis confirmed that endoscopic therapy reduces rebleeding risk (Forrest type Ia, Ib, IIa) compared with intravenous PPIs alone (OR 0.56; 95% CI 0.34-0.92) [46]. Another meta-analysis confirmed the value of endoscopic therapy in this high-risk population by showing that PPI treatment only reduces mortality if endoscopic therapy has been done [47]. Lastly, the literature recommends combined endoscopic therapy (epinephrine + clips or thermal devices) and not epinephrine alone [48].

4.In the presence of an adherent clot (Forrest type IIb), endoscopic hemostasis is possible when the clot is small *(strong agreement).*

The usual approach to an adherent clot is to seek to dislodge it by irrigation. When the clot is resistant to washing, reported rebleeding rates range from 0% [49] to 35% [50]. Two meta-analyses showed no advantage of endoscopic therapy over medical therapy [48,51]. In contrast, no data suggestan excess risk related to endoscopic therapy and a systematic review showed a low incidence of complications [52].

5.In the presence of stigmata associated with a high risk of rebleeding (Forrest type Ia, Ib, IIa, IIb), PPI treatment should be continued at “high” doses for 72 h *(strong agreement).*

PPIs have been proposed to prevent early rebleeding after spontaneous or endoscopic hemostasis and several studies suggest they are beneficial at “high” doses (according to the regimen initially proposed with intravenous omeprazole: 80 mg in 30 min, then 8 mg/h for 72 h). The first quality study showed that PPIs at “high” doses, compared with placebo, significantly reduced the risk of recurrent bleeding (hazard ratio 3.9; 95% CI 1.7-9), but no difference in need for surgery or death [53]. A meta-analysis showed that PPI treatment with or without endoscopic therapy, compared with placebo or an H_2_ receptor antagonist, reduced the risk of rebleeding (OR 0.45; 95% CI 0.36-0.57) and the need for surgery (OR 0.56; 95% CI 0.45-0.7), but not mortality [54]. Complementary analyses showed that in patients with active bleeding or visible blood vessels who were receiving hemostatic treatment, a high intravenous dose of PPI reduced rebleeding, surgery, and also mortality (OR 0.57; 95% CI 0.34-0.96) [54]. Two meta-analyses showed no effect on mortality of lower doses of PPI [48,54]. Lastly, a randomized study [55] of esomeprazole (80 mg then 8 mg/h for 72 h) compared with placebo in patients with peptic ulcer bleeding (Forrest type Ia to IIb) who received endoscopic therapy showed a significant reduction in recurrent bleeding at 72 h (5.9% vs. 10.3%) and for up to 30 days [55]. In contrast, there was no advantage to the use of esomeprazole in terms of surgery or all-cause mortality rate [55]. A recent meta-analysis (7 randomized studies, 1,157 patients) comparing high-dose PPI (80 mg of omeprazole or pantoprazole then 8 mg/h for 72 h) with non-high-dose PPI found no difference in the rates of rebleeding, surgical intervention, or mortality [56]. The study did, however, have several limitations: the analysis was not intention to treat, studies of insufficient power were included, and quite high doses of PPI were in fact administered in the non-high-dose groups. Lastly, cost-effectiveness analyses showed that high-dose PPIs were beneficial after successful endoscopic hemostasis [17,57].

6.Second-look EGD should not be done routinely *(strong agreement).*

7.Second-look EGD probably should be done when a high-risk stigmata has been observed *(weak agreement).*

Second-look EGD is scheduled for 16 to 24 h after the initial endoscopy. A 2010 meta-analysis evaluated the value of second-look endoscopy with thermal coagulation or injection of adrenaline [58]. Thermal coagulation reduced recurrent bleeding (4.2% vs. 15.7%, relative risk 0.29; 95% CI 0.11-0.73) but had no effect on need for surgery or mortality. Second-look endoscopy with injection of adrenaline was not associated with any benefit. A meta-analysis done for the 2010 international consensus conference [2] suggested that second-look endoscopy reduces the risk of rebleeding (OR 0.59; 95% CI 0.38-0.91) and surgery (OR 0.43; 95% CI 0.19-0.96), but not mortality. Second-look endoscopy reduced rebleeding by 33% (*p*=0.03) in a study in which 47% of the patients were in a state of shock [59]. Saeed et al. showed in patients at high hemorrhagic risk according to the Forrest classification, a reduction in rebleeding from 24% to 0% (*p*< 0.05) [60]. Two meta-analyses showed a significant reduction in hemorrhagic risk (respectively, relative risk 0.29 and OR 0.59) in at-risk patients [46,61]. The cost-effectiveness ratio of a strategy involving routine second-look EGD has not yet been assessed.

8.Patients with ulcer bleeding should not be treated with H_2_ receptor antagonists *(strong agreement).*

A 1985 meta-analysis of 27 studies of H_2_ receptor antagonists suggested that they marginally reduced mortality and surgery, in particular in patients with a gastric ulcer [62]. This was confirmed by a more recent meta-analysis [63]. Following the June 16, 2011 meeting of the French Transparency Commission, it was concluded that H_2_ receptor antagonists are of little therapeutic benefit in peptic ulcer-related bleeding.

9.In adults, in case of Forrest type Ia and Ib, first-intention selective arterial embolization by interventional radiology should probably be used following failure of endoscopic therapy *(weak agreement).*

10.In adults, in case of Forrest type Ia and Ib and catastrophic bleeding, first-intention surgical hemostasis should probably be used following failure of endoscopic therapy if local conditions do not allow arterial embolization *(strong agreement).*

If endoscopic therapy fails, percutaneous transcatheter arterial embolization is an alternative to surgery, notably in patients at a high surgical risk. A recently published analysis of 35 studies and 927 patients showed that the technical and clinical success rates of embolization ranged from 52% to 100% and 44% to 100%, respectively [64]. Rebleeding was observed in 0 to 55% of cases depending on the study. Successful embolization improved survival 13.3-fold [64]. Retrospective comparisons between surgery and embolization showed equivalent clinical success and mortality, although embolization was applied to an older population with a higher prevalence of comorbidities [64].

11.Biopsy screening for *Helicobacter pylori* infection can be performed during the first EGD for GI bleeding without worsening the bleeding *(strong agreement).*

No study has evaluated the hemorrhagic risk linked to gastric biopsy during upper GI bleeding. In a prospective, case–control study, gastric biopsies were performed to screen for *Helicobacter pylori* in 324 patients with GI bleeding and 164 patients with uncomplicated ulcer [65]. The hemorrhagic risk in the group admitted for GI bleeding was not increased [65]. However, the sensitivity of the rapid urease test was significantly lower in this group (false-negative 16.7% vs. 5.6%) [65].

12.There is probably no advantage to emergency treatment of *Helicobacter pylori* infection in the case of GI ulcer bleeding *(strong agreement).*

A meta-analysis clearly established that eradication therapy of *Helicobacter pylori* infection compared with “conventional” (OR 0.17; 95% CI 0.1-0.32) or “prolonged” (OR 0.25; 95% CI 0.08-0.76) antisecretory therapy, reduced the long-term (1 year or more) risk of recurrent bleeding [66]. In contrast, no study has shown that eradication therapy is useful in early rebleeding.

13.Aspirin antiplatelet therapy should probably be maintained in the case of GI ulcer bleeding until consultation with specialists *(weak agreement).*

14.In dual antiplatelet therapy, clopidogrel should probably be stopped in the case of ulcer bleeding until consultation with specialists *(strong agreement).*

A meta-analysis showed that discontinuing or not adhering to aspirin given preventively was associated with a threefold higher risk of major adverse cardiac events [67]. Stoppage of antiplatelet treatment in a hemorrhagic setting probably yields no immediate benefit because an antithrombotic effect persists for 7 to 10 days [68]. A randomized study in patients with aspirin-induced peptic ulcer suggested that early reintroduction of aspirin or clopidogrel was possible [69]. Another randomized study in 156 patients with aspirin-induced ulcer bleeding who received endoscopic therapy and PPI treatment showed that immediate reintroduction of aspirin was associated with a twofold higher risk, albeit not statistically significant, of rebleeding [70]. Conversely, discontinuation of aspirin was associated with a significant increase in mortality at 8 weeks [70].

### Area 3: Treatment of upper GI bleeding related to portal hypertension

1.Endoscopic therapy of bleeding esophageal/gastric varices should be done during initial EGD *(strong agreement).*

2.Endoscopic therapy of bleeding esophageal varices is based on band ligation. Sclerotherapy is an alternative in the very young child *(strong agreement).*

3.Endoscopic therapy of bleeding gastric varices is based on obturation with tissue adhesives *(strong agreement).*

EGD is the key examination in management of GI bleeding in cirrhotic patients [5]. No study indicates the best timing, but international recommendations advise as soon as possible (within 12 h) in a resuscitated patient [5]. It is now clear that the treatment of choice in esophageal varices is band ligation, which controls active bleeding and reduces the rebleeding rate with fewer adverse effects than sclerosis [71]. Obturation using cyanoacrylate glue, which is technically more difficult than ligation, is the reference treatment of bleeding gastric varices [72].

4.Vasoactive treatment (terlipressin or somatostatin or somatostatin derivative) should be continued for 3 to 5 days after endoscopic therapy of esophageal/gastric varices rupture *(strong agreement).*

Combined (vasoactive and endoscopic) treatment is more effective than endoscopic therapy in controlling bleeding from esophageal/gastric varices. No clear reduction in mortality by a combined treatment has been demonstrated. The length of vasoactive treatment has not been evaluated in dedicated trials. Sung et al. observed significantly less recurrent bleeding at 48 h in patients treated by ligation and octreotide compared with in those treated by ligation alone [37]. The combination of sclerotherapy and somatostatin for 5 days was more effective than sclerotherapy alone [73]. Besson et al. combined sclerotherapy and octreotide (25 μg/h) for 5 days and observed increased survival without rebleeding 5 days after sclerotherapy [36]. In another study, survival without rebleeding at 5 days was greater in the group of patients treated with endoscopy and vapreotide than in the group treated with endoscopy and placebo [74].

5.In adults, after endoscopic hemostasis of bleeding related to portal hypertension, the placement of a transjugular intrahepatic portosystemic shunt (TIPS) within 72 h should be considered in high-risk patients (Child-Pugh class B with active bleeding and Child-Pugh class C) *(strong agreement)*.

Two studies showed that early placement of TIPS reduces the risk of failure to control bleeding and rebleeding in patients at high risk of recurrence [75,76]. In the more recent study, high-risk patients were defined as Child-Pugh class B patients with persistent bleeding at the time of EGD or Child-Pugh class C patients [76]. Improvement of survival following TIPS placement also was demonstrated compared with the control group (13% vs. 39%) [76].

6.Esophageal/gastric balloon tamponade should be considered after failure of endoscopic therapy pending radical treatment of portal hypertension. In children, its use should probably be envisaged if emergency EGD is not possible *(strong agreement).*

The Sengstaken-Blakemore tube (with two balloons for esophageal varices) and the Linton-Nachlas tube (single balloon for gastric cardia varices or fundal varices) were tested in a 1988 old studies for tamponade of bleeding varices. The success rate was 40% to 90%, but there were numerous complications (inhalation lung disease, esophageal ulceration, and rupture) and rebleeding occurred in approximately one of two cases [77].

7.Antibiotic prophylaxis with third-generation cephalosporin or with fluoroquinolone for 5 to 7 days should be given to any cirrhotic patient with GI bleeding *(strong agreement).*

A bacterial infection is noted in approximately 40% of cirrhotic patients in the 7 days following their admission for upper GI bleeding [78]. These infections are independently associated with the risk of rebleeding and with mortality [79]. A meta-analysis has clearly established that prophylactic antibiotic therapy significantly reduces infection (by 32%; 95% CI 22–42) and death (by 9%; 95% CI 2.9-15.3) [80]. The oral administration of a quinolone is recommended in most patients [5,81]. Intravenous quinolone is possible if the oral route is not available. A recent study in a population of patients with advanced cirrhosis (Child-Pugh class B or C) showed that intravenous ceftriaxone was associated with a lower infection rate than oral norfloxacin [82]. International recommendations state that intravenous ceftriaxone should be considered as prophylactic antibiotic therapy in this population [5].

8.Lactulose treatment to prevent hepatic encephalopathy should probably not be initiated during GI bleeding in a cirrhotic patient (*expert opinion, strong agreement*).

9.In adults, PPI therapy should not be initiated or continued when EGD has confirmed a diagnosis of ruptured esophageal/gastric varices (*strong agreement*).

10.In children, PPI therapy should probably be initiated or continued in case of esophageal/gastric varices rupture *(weak agreement).*

Esophageal ulceration is a possible complication of variceal ligation. In a randomized, controlled trial, PPI therapy reduced the size, but not the number, of postligation ulcers [83]. Several retrospective studies suggest that PPIs may increase the risk of bacterial infection in this population [84]. In children, however, gastritis is frequently associated with portal hypertension [85].

11.One objective of hemodynamic treatment during esophageal/gastric varices rupture should be to restore a satisfactory mean blood pressure to preserve tissue perfusion *(strong agreement).*

12.In adults, during esophageal/gastric varices rupture, early hemodynamic treatment should probably maintain mean blood pressure at approximately 65 mmHg in most patients *(strong agreement).*

In cirrhosis, portal pressure seems to be related linearly to plasma volume and may affect the severity of bleeding [86]. The target mean blood pressure is not well known in this population. On the basis of extrapolation of recommendations established for hemorrhagic shock in trauma patients [87], but also for septic shock [88], a mean blood pressure of approximately 65 mmHg can be proposed.

13.Blood transfusion in most patients should probably target a hemoglobin concentration of 7 to 8 g/dL *(strong agreement).*

The target hemoglobin concentration in esophageal/gastric varices rupture is not clearly defined. In trauma patients with hemorrhagic shock, a target hemoglobin concentration of 7 to 9 g/dL has been proposed [87]. Restricted blood transfusion (hemoglobin concentration 7 to 8 g/dL) also is recommended in the specific context of GI bleeding related to portal hypertension [5].

14.In GI bleeding in patients with cirrhosis, there is probably no indication for administration of fresh-frozen plasma with the objective to correct a coagulopathy *(strong agreement).*

15.In GI bleeding in patients with cirrhosis, there is no indication for administration of fresh-frozen plasma before EGD *(strong agreement).*

In hemorrhagic shock due to trauma, early treatment with fresh-frozen plasma is recommended when bleeding is massive [87] but is much debated in cirrhotic patients [5]. An association between this type of transfusion and the risk of excessive plasma volume increase and worsening of portal hypertension has been suggested [10]. Lastly, prothrombin time and international normalized ratio are not good indicators for coagulability in patients with cirrhosis and attempts to correct them are therefore not recommended [5].

16.In GI bleeding in patients with cirrhosis, there is no indication for the administration of factor VIIa *(strong agreement).*

A recent randomized trial in patients with cirrhosis and variceal bleeding found no difference between factor VIIa and placebo in terms of control of bleeding or survival [89].

17.In GI bleeding in patients with cirrhosis, platelet transfusion should probably be considered when bleeding is uncontrolled and platelet count is <30,000/mm^3^*(strong agreement).*

Platelet transfusion in severe bleeding is usually recommended when platelet count is <50,000/mm^3^[87,90]. No study has examined this question in the particular setting of GI bleeding in the cirrhotic patient. The risk of worsening portal hypertension has been raised [10]. Thrombocytopenia is common in the cirrhotic patient and is a poor indicator of hemorrhagic risk [5]. On the basis of these arguments, a transfusion is usually recommended for a platelet count <30,000/mm^3^ but should not delay endoscopy [5,10].

18.In adults, in esophageal/gastric bleeding, beta-blocker treatment should be started when vasoactive treatment is discontinued *(strong agreement).*

Secondary prophylaxis of bleeding from esophageal/gastric varices is based on the combination of noncardioselective beta-blockers and repeated ligation [5]. Early introduction of beta-blockers seems to avoid a rebound effect of portal hypertension favoring rebleeding. A recent meta-analysis of 23 studies showed that combined treatment (beta-blocker and ligation) significantly reduced rebleeding in comparison with endoscopic therapy alone (OR 0.68; 95% CI 0.52-0.89) or pharmacological treatment alone (OR 0.71; 95% CI 0.59-0.86) [91]. The median interval to introduction of beta-blockers was 3 (range 1–7) days [91].

19.After ligation of esophageal varices, nasogastric intubation should probably be avoided *(expert opinion, strong agreement).*

### Area 4: Management of presumed lower GI bleeding

1.In adults with massive hematochezia, demonstration of active bleeding by abdominal CT angiography or arteriography justifies embolization as first-line therapy *(strong agreement).*

After angiographic localization of bleeding, the hemostatic efficacy of embolization is approximately 90%. One study showed, however, that embolization was associated with an approximately 10% risk of recurrent bleeding and more rarely a risk of colonic ischemia [92]. In approximately 5% of cases, embolization was technically impossible [92].

2.In massive hematochezia, and in the absence of detectable bleeding on abdominal CT angiography or arteriography (or scintigraphy in children), a prepared colonoscopy should be performed within 24 h *(strong agreement).*

3.In massive and persistent hematochezia, and in the absence of detectable bleeding on abdominal CT angiography or arteriography, a prepared colonoscopy should probably be done within 12 h with the objective of performing endoscopic hemostasis *(strong agreement).*

Colonoscopy identifies the cause of hematochezia in approximately 75% of cases [93]. Colonoscopy after preparation is therefore recommended within 24 h [25,94]. If hematochezia persists, colonoscopy within a shorter timeframe (12 h) should be considered, notably with a view to hemostatic treatment [95-100]. Initial endoscopic hemostasis is successful in nearly 95% of cases [93,94,101,102]. In contrast, the results in terms of definitive hemostasis vary from one study to another. In all these studies the risk of complications of colonic endoscopic hemostasis was low or zero.

4.In adults with massive hematochezia, rectosigmoidoscopy probably should be done if full colonoscopy cannot be performed within 24 h *(strong agreement).*

In the 80% of cases when bleeding ceases spontaneously, a prepared colonoscopy should be performed as soon as possible [103]. Early colonoscopy increases the chances of identifying the cause of bleeding and of proposing endoscopic hemostasis [93,95,96,98,99]. In contrast, the prognostic value of early colonoscopy seems more questionable [96]. Rectosigmoidoscopy, often performed in imperfect conditions in terms of preparation and safety, rarely dispenses with the need for full colonoscopy [25,94].

5.When lower GI bleeding is massive, surgical hemostasis should be proposed in case of arterial embolization or colonoscopy failure or rebleeding *(strong agreement).*

6.When lower GI bleeding is catastrophic, surgical hemostasis should be performed if arterial embolization is not possible under local conditions *(strong agreement).*

The indications for surgical hemostasis have been considerably reduced because of advances in embolization and in endoscopic therapy and are limited to failure of embolization or of colonoscopy or of both [25,94]. Such situations essentially concern diverticular bleeding. If there is an indication for surgery, an attempt should be made to determine the site of bleedingin order to adapt the surgical procedure. When the site of bleeding is known with certainty, partial colectomy should be performed. In diffuse colonic diverticulosis when the site of bleeding is unidentified, subtotal colectomy should be preferred.

7.In massive or persistent lower GI bleeding, the small intestine should be examined as soon as possible when CT angiography and colonoscopy fail to locate the source of bleeding *(strong agreement).*

When the cause of bleeding is unknown, small intestine involvement should be considered whenever terminal ileoscopy (which should be routine during colonoscopic examination of lower GI bleeding) reveals blood or mucosal anomalies [104]. Etiological diagnosis of small intestine bleeding often is difficult [105]. Endoscopic techniques (capsule endoscopy, enteroscopy) usually enable diagnosis [105]. The data on endoscopic hemostasis of bleeding in the colon can be transposed to small intestine lesions accessible to endoscopy, albeit with a probably higher risk of complications.

### Area 5: Prevention of upper GI bleeding in intensive care

1.Patients with a history of peptic ulcer admitted to intensive care probably should be considered at risk of GI bleeding *(strong agreement).*

A 1983 retrospective analysis of patients admitted to medical-surgical intensive care unit defined a risk score for upper GI bleeding in intensive care as the sum of coefficients associated to 24 risk factors [106]. A history of ulcers was associated with the most determinant coefficient [106], a result confirmed by another study [107]. It also should be emphasized that patients with a peptic ulcer within the previous month or year often are excluded from studies that analyze upper GI bleeding in intensive care [108,109].

2.Early enteral feeding is effective in preventing “stress ulcer” bleeding *(strong agreement).*

3.Patients requiring mechanical ventilation for more than 48 h and for whom enteral feeding is not possible should be considered to be at risk of “stress ulcer” bleeding *(strong agreement).*

4.Patients admitted to intensive care with kidney failure and/or coagulopathy and/or receiving antiplatelet therapy should be considered to be at risk of “stress ulcer” bleeding *(strong agreement).*

5.Routine drug prophylaxis of “stress ulcer” should not be used in intensive care patients with enteral feeding *(strong agreement).*

6.Ulcer prophylaxis medication should probably be given routinely in intensive care patients with a history of peptic ulcer (even if enterally fed) *(weak agreement).*

7.Ulcer prophylaxis medication probably should be given routinely to intensive care patients receiving antiplatelet therapy (even if enterally fed) *(weak agreement).*

8.In the absence of enteral feeding, ulcer prophylaxis medication should probably be given to ventilated patients *(weak agreement).*

9.In the absence of enteral feeding, ulcer prophylaxis medication should probably be given to patients with coagulopathy *(weak agreement).*

Several criteria, including mechanical ventilation [109-111], coagulopathy [109,110], kidney failure [111], and lack of enteral feeding [111], have been identified as risk factors, sometimes independent, for upper GI bleeding in intensive care. In 174 patients, mechanical ventilation for more than 5 days and coagulopathy (defined by platelet count <50 000/mm^3^, effective anticoagulation or lengthening of the partial thromboplastin time) were the risk factors most strongly associated with upper GI bleeding [110]. In a vast prospective and multicenter analysis in 2,252 patients, Cook et al. identified mechanical ventilation of more than 48 h (OR 15.6) and coagulopathy (OR 4.3) as the only two risk factors independently associated with upper GI bleeding [109]. The incidence of bleeding was 8.4% in the presence of these two risk factors and only 0.1% in their absence [109]. Some years later, the same team in a randomized, multicenter trial in 1,077 mechanically ventilated patients compared two ulcer prophylactic drugs (ranitidine and sucralfate) and identified a risk factor independent of upper GI bleeding—peak creatinine (by 100 μmol/L increments, OR 1.16; 95% CI 1.02-1.32)—and two protective factors—enteral feeding (OR 0.3; 95% CI 0.13-0.67) and administration of ranitidine (OR 0.39; 95% CI 0.17-0.83) [111].

10.In children, a pediatric risk of mortality score (PRISM) >10 associated with respiratory failure or coagulopathy or both probably calls for ulcer prophylaxis *(strong agreement).*

Pediatric data enabling analysis of the benefits of prophylactic treatment of “stress ulcer” bleeding are rare [112]. A 1998 prospective study in 881 children identified 3 risk factors independent of clinically significant upper GI bleeding acquired in intensive care: respiratory failure (OR 2.87; 95% CI 1.3-82.8). coagulopathy (OR 9.3; 95% CI 2.8-30.3), and a pediatric risk of mortality score ≥10 (OR 13.4; 95% CI 3.7-47.9) [113]. The presence of two or more factors was associated with an approximately 90% incidence of GI bleeding. This study confirms other data identifying mechanical ventilation, lung disorders, and thrombocytopenia as risk factors for GI bleeding [114-116].

11.Screening for *Helicobacter pylori* should not be routine in intensive care patients *(strong agreement).*

The prevalence of *Helicobacter pylori* infection in intensive care is greater than that in a control population [117]. However, there are few arguments to suggest that *Helicobacter pylori* is responsible for upper GI bleeding in intensive care. Three studies have reported a statistical link between *Helicobacter pylori* infection and stress-related GI bleeding [118-120]. Data from two of them differ according to the tests used to diagnose *Helicobacter pylori* infection [118,119]. By coupling several diagnostic methods (serology, biopsy, enzyme immunoassay detection of antigen in stools), Maury et al. found a significantly greater incidence of *Helicobacter pylori* infection in the case of GI bleeding (36% vs. 16%) [120]. However, this study was conducted in only 25 patients [120]. Lastly, three other studies found no correlation between *Helicobacter pylori* infection and “stress ulcer” bleeding [117,121,122].

12.A nasogastric tube should probably be removed once it is no longer used *(expert opinion, strong agreement)*.

13.A large bore nasogastric tube for aspiration should probably be replaced by a small-calibre enteral tube as soon as possible *(expert opinion, strong agreement)*.

14.Antacids should not be used to prevent “stress ulcer” bleeding *(strong agreement).*

15.H_2_ receptor antagonists and PPIs are probably comparable but of low efficacy in preventing “stress ulcer” bleeding *(weak agreement).*

16.H_2_ receptor antagonists and PPIs are probably comparable regarding the risk ventilator-associated pneumonia during mechanical ventilation *(strong agreement).*

In the prevention of upper GI bleeding in intensive care, H_2_ receptor antagonists, long used in clinical trials, are superior to placebo [3,123], antacids [123], and sucralfate [124]. A recent meta-analysis showed that H_2_ receptor antagonist treatment was associated with a significant reduction in the risk of GI bleeding compared with placebo (OR 0.47; 95% CI 0.29-0.76) [3]. In contrast, this risk decrease was not observed in the subgroup of enterally fed patients (OR 1.26; 95% CI 0.43-3.7) [3]. PPIs have not been compared with placebo in this indication. A randomized, controlled study in 359 mechanically ventilated patients compared a PPI (oral omeprazole) and an H_2_ receptor antagonist (intravenous cimetidine) in prophylaxis of upper GI bleeding in intensive care [125]. The time spent at pH ≥6 was significantly greater in the omeprazole group than in the cimetidine group (100% vs. 50% per treatment day), and the prevalence of GI bleeding did not differ between the two groups. A recent meta-analysis comparing PPIs and H_2_ receptor antagonists confirmed this result [126] and also showed that there was no difference between PPIs and H_2_ receptor antagonists in terms of the risk of nosocomial pneumonia [126].

## Competing interests

Scientific presentation at a symposium held on by W.L GORE: Christophe Bureau, Nicolas Carbonell, Dominique Thabut. Scientific presentation at a symposium held on by Eumédica: Dominique Pateron, Nicolas Carbonell. Participation in an observatory supported by Eumédic: Nicolas Carbonell, Dominique Thabut. Expert’s report for Sanofi-Aventis and Ethypharm: Marc Bardou. All other authors declare that they have no conflicts of interest.

## Authors’ contribution

DO, MD, DS and CG participated in the design and the coordination of the study and checked compliance to the GRADE method. CG and the group of experts participated in the development and the grading of the level of proof of the recommendations. DO carried out the RAND/UCLA method for the collective scoring. DO, MD, DS, CG and the group of experts participated in the redaction of the arguments’ recommendations. All authors read and approved the final manuscript.

## Authors’ information

^1^Organising Committee for the SRLF Recommendations and Assessments Board.

^2^Expert coordinator.

^3^Experts: Marc Bardou (Dijon), Sophie Branchereau (Le Kremlin-Bicêtre), Christophe Bureau (Toulouse), Nicolas Carbonel (Paris), Philippe Cluzel (Paris), Emmanuel Guérot (Paris), Pierre-François Laterre (Bruxelles), Gilles Lesur (Boulogne), Emmanuel Mas (Toulouse), Eric Maury (Paris), Stéphane Nahon (Montfermeil), Philippe Otal (Toulouse), Dominique Pateron (Paris), Gaël Piton (Besançon), Jean-Pierre Quenot (Dijon), Marika Rudler (Paris), Dominique Thabut (Paris), Pierre Tissières (Le Kremlin-Bicêtre), Patrice Valleur (Paris)

SRLF Recommandations and Assessments Board: Cédric Bretonnière, Karim Chaoui, Aurélie Cravoisy, Michel Djibré, Laurent Dupic, Fabienne Fieux, Dominique Hurel, Virginie Lemiale, Olivier Lesieur, Martine Lesny, Pascal Meyer, Christophe Milési, Benoit Misset, Mehran Monchi, David Orlikowski, David Osman, Jean-Pierre Quenot, Daniel Da Silva, Lilia Soufir, Thierry Van Der Linden, Isabelle Verheyde.
